# Influence of supporting teeth quantity of surgical guide on the accuracy of the immediate implant in the maxillary central incisor: an in vitro study

**DOI:** 10.1038/s41405-024-00292-7

**Published:** 2024-12-28

**Authors:** Meo Nguyen, Huynh Kim Khanh Nguyen, Thien Nga Nguyen, Nam Cong-Nhat Huynh

**Affiliations:** Faculty of Odonto-Stomatology, University of Medicine and Pharmacy at Ho Chi Minh City, Ho Chi Minh City, 749000 Vietnam

**Keywords:** Dental implants, Dental treatment planning

## Abstract

**Introduction:**

Guided surgery for immediate anterior implants aims to reduce the time required for aesthetic and functional immediate loading. However, the limited surface area of anterior teeth for guide stabilization may affect the accuracy of implant positioning. This in vitro study evaluated the effect of the number of supporting teeth on the accuracy of immediate implants in the maxillary central incisor region.

**Methods:**

28 replica implants were inserted into 28 upper jaw models, simulating immediate post-extraction sockets of tooth 11. Based on the number of supporting teeth, the implants were categorized into G1 (four adjacent teeth) and G2 (six adjacent teeth). The planned and actual implant positions were compared using the evaluation module of the implant planning software. Angular and 3D deviations were measured as the primary outcomes. Statistical analysis was performed using the two-sample t-test, with *p*-values less than 0.05 defined as statistically significant.

**Results:**

Between group G1 and G2, angular deviation was measured at 4.63 ± 0.71° and 3.59 ± 0.97°, respectively, while the implant apex 3D deviation was 2.08 ± 0.21 mm for G1 and 1.40 ± 0.27 mm for G2. These differences were statistically significant (*p* = 0.003 and *p* < 0.001, respectively). Other discrepancy variables in G2 demonstrated lower values but were not statistically significant compared to G1.

**Conclusion:**

The number of supporting teeth for the surgical guide can influence the accuracy of immediate implant surgery. While both four-teeth and six-teeth supports demonstrated acceptable clinical implant accuracy, a surgical guide supported by six teeth can enhance implant precision.

## Introduction

The restoration of the anterior region requires high aesthetic outcomes, and immediate implant placement is commonly indicated in this area to meet aesthetic demands, as it allows for provisional fixed prostheses to be connected to newly inserted implants, thereby achieving optimal esthetic results [1–4]. However, the technique of immediate implant surgery is complex. It presents several challenges, such as the risk of inadequate primary stability and drill sliding during the implant bed preparation [5, 6]. Drill slippage may occur due to the tendency of the drill to move toward less resistant areas, such as the tooth socket, or when the bur contacts the wall at a flat angle, which can compromise the correct positioning of the implant [7].

The three-dimensional (3D) implant position is crucial for the long-term success of the implant [8]. This position is planned prior to surgical placement and is determined by the design of the prosthesis and the anatomy of the placement site. Proper implant positioning is essential for achieving aesthetic outcomes and ensuring the expected primary stability for an immediate implant [9, 10]. Facial malpositioning of the implant often leads to mucosal recession around the immediate implant, resulting in aesthetic complications in the aesthetic region [11].

Static computer-assisted implant surgery (sCAIS) can assist surgeons in accurately transferring planned implant positions from virtual planning to surgical placement [12]. Recent studies have demonstrated that sCAIS, which employs surgical guides to facilitate implant placement, can minimize discrepancies between the planned and actual implant positions compared to freehand surgery [13, 14]. Surgical guide systems can help stabilize the drill during the procedure, reduce deviations, and enhance treatment outcomes [15]. Therefore, this surgical technique contributes to a decreased risk of complications arising from inaccuracies, ultimately leading to improved aesthetic and functional results [16, 17].

Guided surgery can be accurate and predictable for implant placement. However, errors at each step can significantly impact accuracy and lead to potential deviations from the ideal implant position. Therefore, it is crucial to identify and understand the possible risks for deviations in the sCAIS procedure [18]. One source of error is the stabilization of the surgical guide, which is influenced by the number of supporting teeth and can affect the accuracy of surgical guides [19]. An in vitro study investigating the influence of surgical guide support demonstrated that guides supported by four adjacent teeth were as accurate as those supported by the entire arch in single-tooth gap situations [20]. The findings from this study suggest that guides supported by posterior teeth were more accurate than those supported by anterior teeth due to their increased surface area. Additionally, the accuracy of immediate implant placement was found to be lower than that of implants placed in healed sockets. A recent in vitro study also indicated that shortened arch surgical guides supported by four teeth were more accurate in a single edentulous space compared to full arch surgical guides [21].

However, there is still limited evidence in the literature comparing the number of supporting teeth for immediate implant surgery. Given the common indication for immediate implantation in the anterior region, where teeth have smaller surface areas than those in the posterior region, there is a potential for increased inaccuracies when using a short arch-supported surgical guide.

Therefore, the aim of this study was to evaluate the influence of the number of supporting teeth on the accuracy of implants placed using surgical guides in immediate implant surgery in the anterior upper jaw region, utilizing study models that simulated upper right central incisor extraction sockets.

## Materials and methods

### Study design

**Fig. 1:**
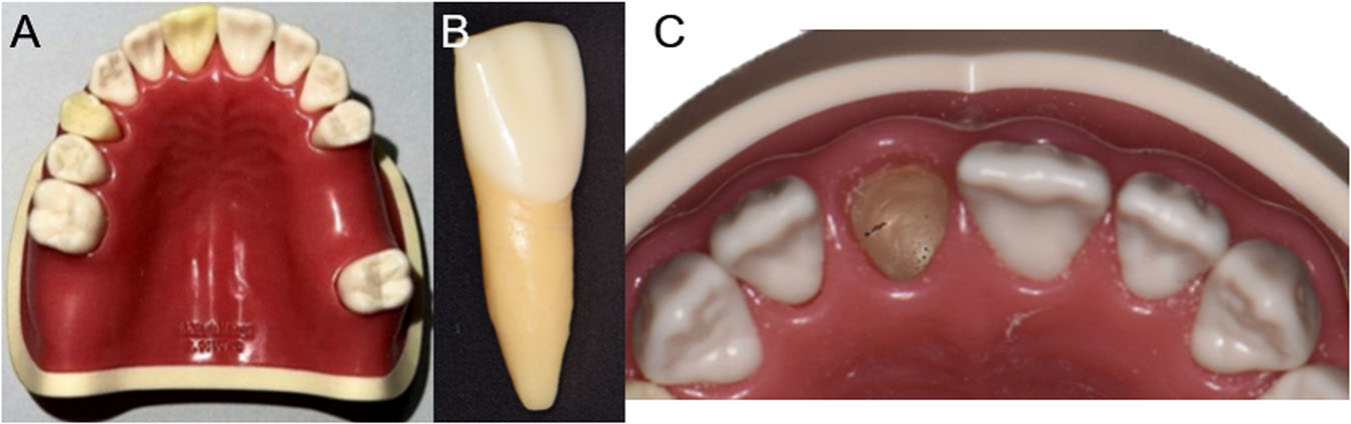
The maxillary model. The maxillary model used in the study (**A**), and removable tooth 11 (**B**) that simulates extracted tooth socket for immediate implant (**C**).

Twenty-eight acrylic resin upper jaw models (BASIC-DY. K-BU, M.Tech Korea, Korea) were utilized in this study, with the alveolar bone constructed from resin to simulate the hardness of human bone with bone density classified as D2 according to Misch classification (Fig. 1). Each model featured a removable tooth 11, which simulates an extracted alveolar socket for immediate implant surgery in the anterior region.

The sample size was calculated based on the implant apex deviation value reported by El Kholy et al. [20], using a type I error (α) of 0.05 and a power of 0.80 to determine the number of implants required for statistical significance. Considering a potential 10% data loss for each group, the study concluded that 28 implants would be sufficient.

### Implant planning

A cone-beam computed tomography (CBCT) system (Orthophos SL 3D, Dentsply Sirona, USA) was used to scan each study model, producing a Digital Imaging and Communication in Medicine (DICOM) file (60 kVp, 3 mA, voxel size 80 µm, field of view 8 × 8 cm). The models were then scanned by an intraoral scanner (TRIOS 4, 3-Shape, Denmark) to obtain a Standard Tessellation Language (STL) file. Implant planning was conducted using coDiagnostiX version 10.6 software (Dental Wings, Germany), which allowed importing the DICOM and STL files. These files were superimposed using anatomical landmarks of the teeth.

The current crown of tooth 11 served as a reference for the prosthetic design. The digital implant position was planned for each sample based on both the prosthetic requirements and the anatomy of the placement site. The implant utilized in this study was a bone-level tapered implant measuring 4.1 mm in diameter and 16 mm in length (Straumann AG, Basel, Switzerland), providing sufficient apical bone to achieve primary stability for immediate implantation [22]. All implants were positioned to meet the anatomic and prosthetic criteria outlined by Buser [10] for maxillary anterior implants. An experienced clinician performed all the implant planning.

### Surgical guide fabrication

The surgical guides were designed using the same software based on the planned implant position. Different surgical guides were created for each group, depending on the number of supporting teeth during implant placement. For the four-teeth group (G1), the surgical guides were supported by four adjacent teeth (teeth number 13, 12, 12, 21). In contrast, for the six-teeth group (G2), the surgical guides were supported by six adjacent teeth (teeth numbers 14, 13, 12, 21, 22, 23). Metal cylindrical sleeves measuring 5 mm in diameter, 5 mm in height, and 4 mm in length from the implant crest (H4) were inserted into the surgical guides. Both groups of surgical guides featured an inspection window at the contact area between teeth 21 and 22 to ensure precise fitting. The offset between the surgical guide and the tooth surface was 0.05 mm. All surgical guides were 3D printed with a thickness of 2.5 mm using medical-grade resin material (DentaGUIDE, Asiga, Australia) with a digital light processing 3D printer (MaxUV, Asiga, Australia). An experienced technician performed all surgical guide fabrication.

### Surgical protocol

**Fig. 2:**
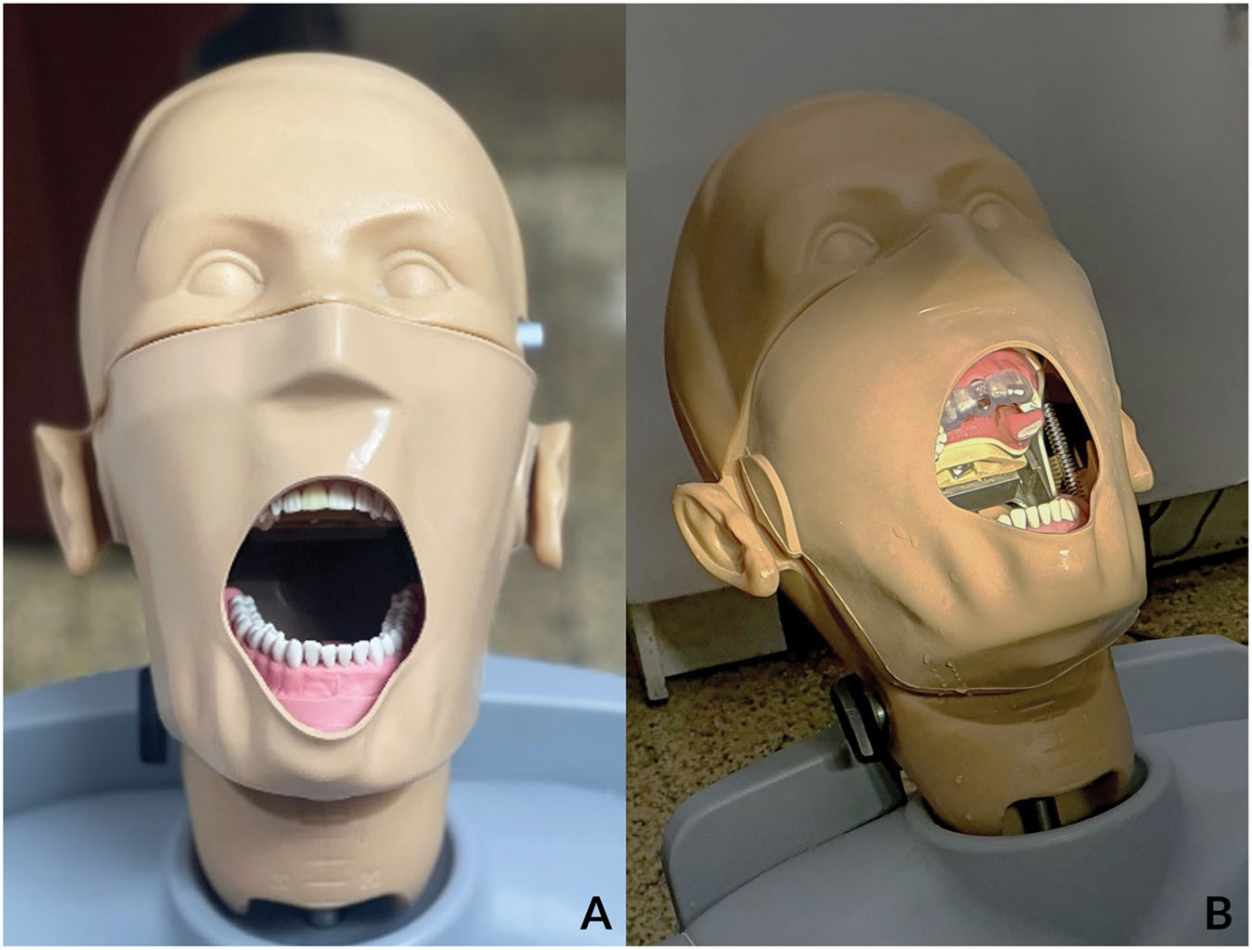
The phantom head simulator unit simulated the surgery procedure. **A** Dental models attached to the phantom head simulator unit simulate the clinical setting. **B** A dental model with tooth 11 removes attach with a surgical guide supported by four adjacent teeth.

The dental models were attached to the phantom head simulator unit to replicate the clinical setting (Fig. 2A). Tooth 11 was removed from the study model, and the surgical guides were then inserted. The fit and stability of the surgical guides were verified through the inspection window, ensuring that the incisal edges of teeth 21 and 22 contacted the inner surface of the surgical guide (Fig. 2B). An experienced surgeon performed all implant placements using the surgical guide. The drilling protocol adhered to the fully guided protocol following the manufacturer’s recommendations, with all implants achieving an insertion torque between 35 and 50 Ncm.

### Accuracy measurements

**Fig. 3:**
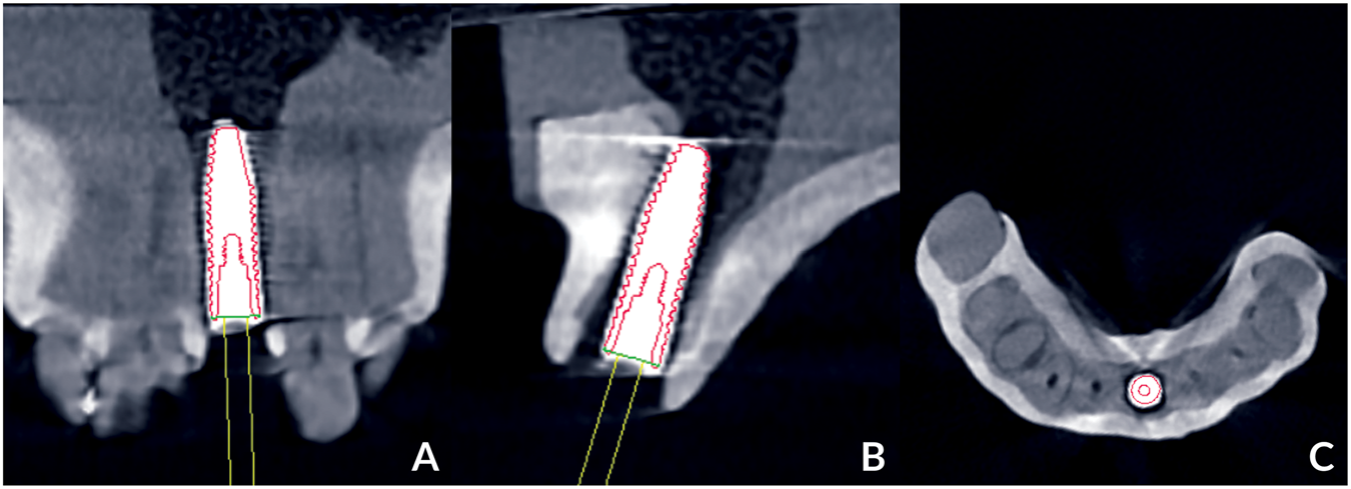
Locating the actual implant position in the “Treatment evaluation” module with CBCT slides in 3 planes. **A** Tangential plane, **B** Cross-sectional plane, **C** Axial plane.

After implant placement, cone-beam computed tomography (CBCT) was utilized to scan all study models using the same parameters as before the surgery. The DICOM file was imported into coDiagnostiX and superimposed with a preoperative DICOM file using anatomic-based registration. The actual implant positions were then identified by analyzing the CBCT slides in three planes: the tangential plane (Fig. 3A), the cross-sectional plane (Fig. 3B), and the axial plane (Fig. 3C). Using the “Treatment evaluation” application of coDiagnostiX software, the algorithm automatically calculated the angular deviation (in degrees) and the three-dimensional (3D) deviation (in millimeters) at both the implant platform and the apex, comparing the placed and planned positions. The primary outcomes were the angular deviation in degrees and the 3D deviation in millimeters at the implant platform and apex (Fig. 3). The secondary outcomes included linear deviations measured in the mesiodistal, buccopalatal, and apicocoronal directions at the implant platform and apex. All procedures and measurements were conducted by a skilled operator.

### Statistical analysis

The data was entered using Microsoft Excel 365 (Microsoft, Washington, U.S.) and analyzed with JASP software version 0.17.2.1 (University of Amsterdam, North Holland, Netherlands). The Shapiro–Wilk test was employed to assess the normality of the data distribution. Data were presented as means and standard deviations (SD) for normally distributed data and as medians and interquartile ranges (IQR) for data that were not normally distributed. Depending on the normality of the data, either the two-sample t-test or the Mann–Whitney U test was utilized to compare the angular deviation and 3D deviation at both the implant platform and apex, as well as the secondary outcomes between the two groups. A *p*-value of less than 0.05 was considered statistically significant.

## Results

### 3D and angular deviations

The results for three-dimensional (3D) and angular deviations are summarized in Table 1. The mean platform deviation for groups G1 and G2 was 0.90 ± 0.22 mm and 0.79 ± 0.18 mm, respectively. The mean apex deviation for the same groups was 2.08 ± 0.21 mm and 1.40 ± 0.27 mm, respectively. The mean angular deviation for the two groups was 4.63 ± 0.71 and 3.59 ± 0.97 degrees, respectively. The Shapiro–Wilk test confirmed that all data were normally distributed, allowing the use of two-sample t-tests.

**Table 1:** The 3D and angular deviations of the implant in each group.

Group	4-teeth (*n* = 14)	6-teeth (*n* = 14)	*p*
3D deviation at platform (mm)			
Mean ± SD	0.90 ± 0.22	0.79 ± 0.18	0.003
Min-Max	0.49–1.26	0.49–1.03	
Range	0.77	0.54	
3D deviation at apex (mm)			
Mean ± SD	2.08 ± 0.21	1.40 ± 0.27	0.158
Min-Max	1.81–2.41	0.81–1.90	
Range	0.60	1.09	
Angular deviation (degrees)			
Mean ± SD	4.63 ± 0.71	3.59 ± 0.97	<.001
Min-Max	3.70–5.80	1.60–4.80	
Range	2.10	3.20	

**Fig. 4:**
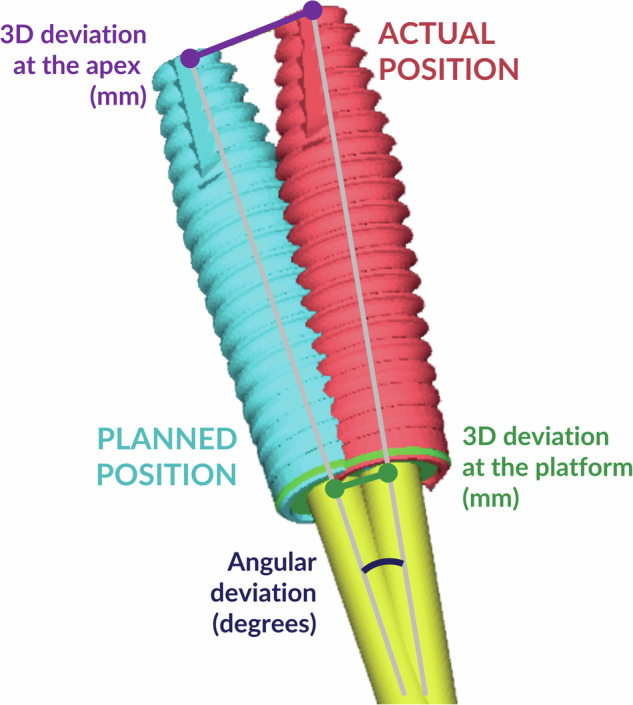
Measurements of the deviation between planned and actual implant position.

There were statistically significant differences between the groups in angular deviation (*p* = 0.003) and 3D deviation at the apex (*p* < 0.001) (see Fig. 4B, C). There was no statistically significant difference between the two groups in 3D deviation at the platform (*p* = 0.158) (see Fig. 4A).

### Linear deviations

The linear implant deviations in three planes (mesiodistal, buccopalatal, and apicocoronal) at both the implant platform and the implant apex are presented in Tables 2 and 3, respectively. These values are expressed in absolute numbers to illustrate the discrepancy distance from the planned implant position, regardless of the direction of deviation. Due to the non-normal distribution of some data, Mann–Whitney U tests were employed. The analyses revealed that only the buccopalatal deviation value at the apex is statically significant between the groups (*p* = 0.001), with the four-teeth group measuring 1.81 [1.47–2.06] mm and the six-teeth group measuring 1.32 [1.05–1.45] mm.

**Table 2:** The overall linear deviations at the implant platform in each group.

Group	4-teeth (*n* = 14)	6-teeth (*n* = 14)	Overall (*n* = 28)
Mesiodisal (mm)			
Median (IQR)	0.17 (0.10–0.21)	0.24 (0.14–0.29)	0.18 (0.13–0.27)
Min-Max	0.01–0.36	0.05–0.36	0.01–0.36
Range	0.35	0.31	0.35
Buccopalatal (mm)			
Median (IQR)	0.56 (0.46–0.93)	0.51 (0.31–0.66)	0.56 (0.41–0.80)
Min-Max	0.39–1.05	0.22–0.96	0.22–1.05
Range	0.66	0.74	0.83
Coronoapical (mm)			
Median (IQR)	0.31 (0.25–0.48)	0.25 (0.16–0.45)	0.27 (0.17–0.49)
Min-Max	0.09–0.79	0.12–0.58	0.09–0.79
Range	0.70	0.46	0.70

**Table 3:** The linear deviations at the implant apex in each group.

Group	4-teeth (*n* = 14)	6-teeth (*n* = 14)	Overall (*n* = 28)
Mesiodisal (mm)			
Mean ± SD	0.45 (0.26–0.74)	0.62 (0.35–0.71)	0.55 (0.31–0.74)
Min-Max	0.16–1.34	0.06–1.05	0.06–1.34
Range	1.18	0.99	1.28
Buccopalatal (mm)			
Mean ± SD	1.81 (1.47–2.06)	1.32 (1.05–1.45)	1.46 (1.30–1.79)
Min-Max	1.25–2.11	0.39–1.65	0.39–2.11
Range	0.86	1.26	1.72
Coronoapical (mm)			
Mean ± SD	0.35 (0.25–0.53)	0.28 (0.20–0.46)	0.32 (0.23–0.52)
Min-Max	0.05–0.87	0.07–0.60	0.05–0.87
Range	0.82	0.53	0.82

**Fig. 5:**
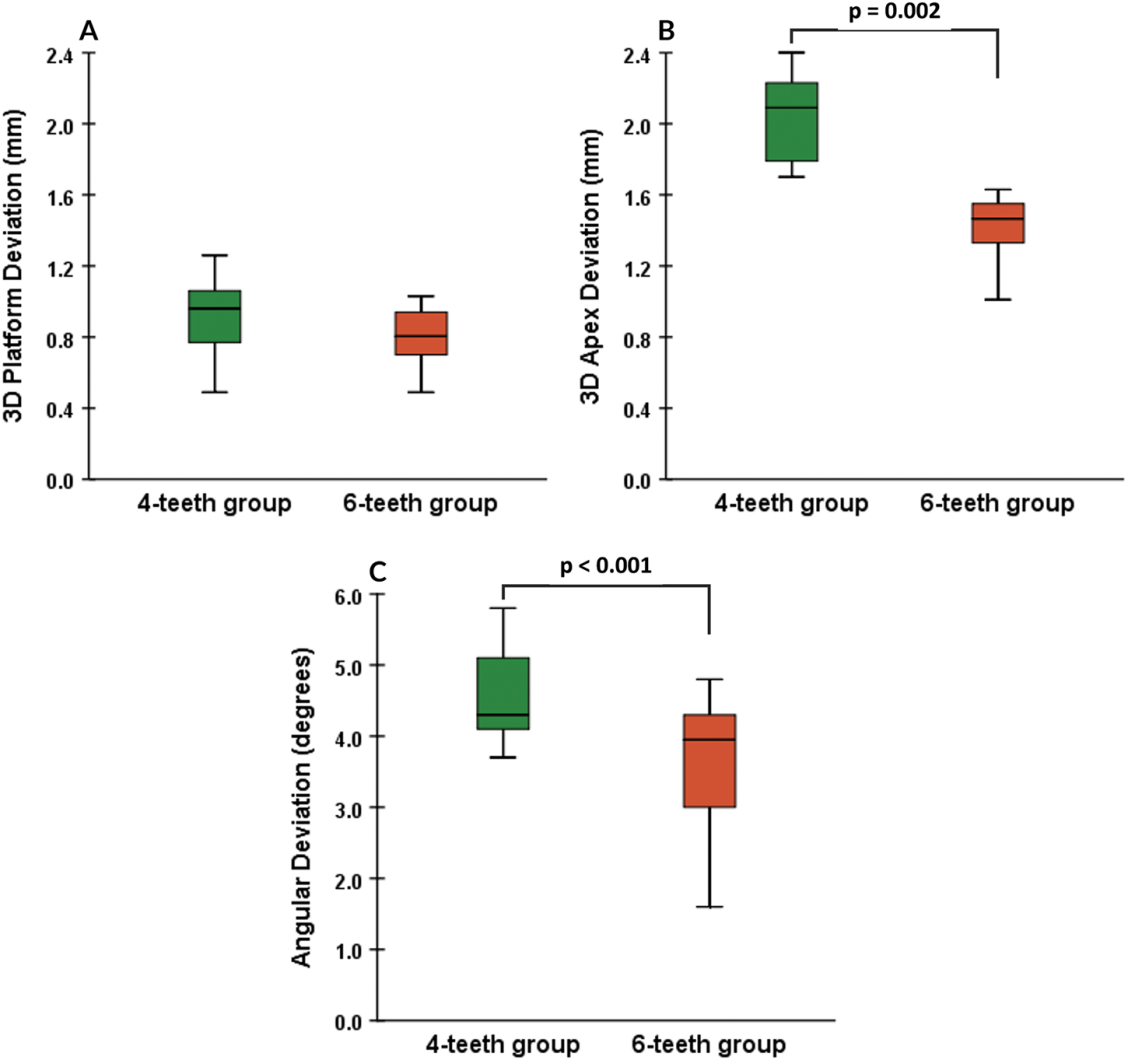
Number of supporting teeth and the 3D deviation. **A** 3D platform deviation. **B** 3D apex deviation. **C** Angular deviation. Two-sample t-tests determined the statistically significant differences between the two groups.

**Fig. 6:**
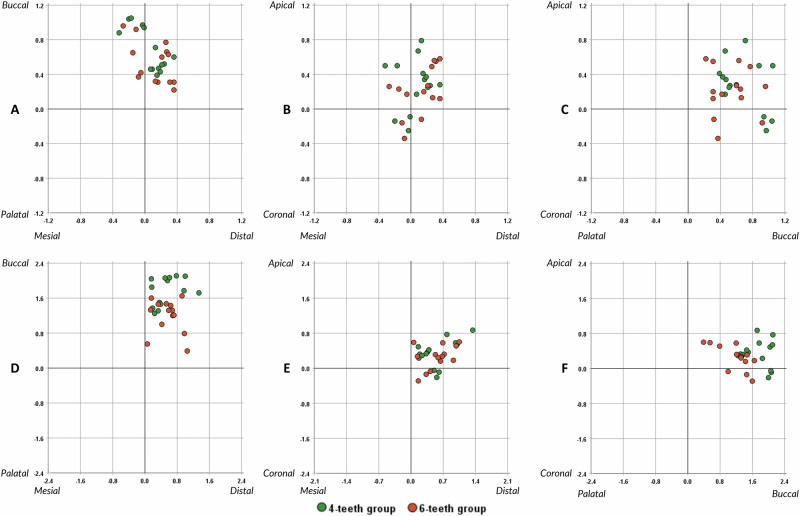
The direction of linear implant deviations at the implant platform and apex. **A** Mesiodistal and buccopalatal planes at implant platform. **B** Mesiodistal and apicocoronal planes at implant platform. **C** Buccopalatal and apicocoronal planes at implant platform. **D** Mesiodistal and buccopalatal planes at implant apex. **E** Mesiodistal and apicocoronal planes at implant apex. **F** Buccopalatal and apicocoronal planes at implant apex.

The two-dimensional scatter plot (Fig. 5) illustrates the direction of linear deviations of implants at both the platform and apex. In both groups, deviations at the implant platform and apex were primarily oriented toward the buccal side (Fig. 6).

## Discussion

This study investigated how the number of teeth supporting surgical guides affects the accuracy of immediate implant placement in a controlled laboratory setting. Significant differences were observed between the two groups in terms of angular deviation and 3D implant apex deviation, with the six-teeth surgical guides demonstrating greater accuracy than the four-teeth support guides. These results indicate that an increase in supporting teeth can positively impact the accuracy of immediate implant placement in the anterior upper region. Despite the differences, both groups achieved clinically acceptable levels of implant positioning accuracy, with deviations measuring less than 2 mm across all variables. A deviation of 2 mm is considered a safe margin when planning implant positions to avoid interference with adjacent anatomical structures [23].

Accurate transfer of the preoperative implant plan to the surgical site is essential for achieving appropriate restoration that ensures both functional and esthetic outcomes, particularly for immediate implant placement in the esthetic zone [4]. Although sCAIS has been shown to offer higher accuracy than freehand implant placement [24], several steps in its procedure could potentially influence implant accuracy, as the overall precision of guided surgery is an accumulation of errors throughout the process [18]. Among the sources of errors, the stabilization of the surgical guide is crucial for the safe and predictable execution of guided surgery. In this in vitro study, despite being conducted in a controlled environment, which could enhance implant accuracy [25], deviation may still arise from factors such as data acquisition using the intraoral scanner and CBCT, surgical guide manufacturing processes, and clinician’s experience [21].

In a systematic review and meta-analysis conducted by Ali Tahmaseb et al. [26], was found that the accuracy of surgical guide placement in partially edentulous cases resulted in a 3D platform deviation of 0.90 mm [0.70–1.00 mm] and an apex deviation of 1.20 mm [1.11–1.20 mm]. The angular deviation was measured at 3.30 degrees [2.07–4.63 degrees]. In comparison, our study exhibited higher deviation values, possibly due to implant placement in extraction sites, which presents a greater risk for deviation. It is assumed that implants placed in such sites tend to deviate towards the side with less bone, which offers reduced mechanical resistance.

The results of the present study conflict with the results of another in vitro study, which compared the effects of different surgical guide designs supported by teeth [21]. Acrylic upper jaws were used with four single-tooth gap situations. The authors found significant differences between surgical guides with 4-teeth support (or shorten-arch) and those with 8-teeth support (or full arch), with the shorter-arch support having lower deviation. These results are discrepant with the present study as although there was a static difference between surgical guides with four-teeth support and six-teeth support; the four-teeth support group had lower accuracy. Though digital impressions for short-span showed higher accuracy than full-arch [27], which could potentially improve the implant accuracy when using surgical guides; the stability of the guides could be more influenced in immediate implants where drills and implants tend to move toward the space of the tooth socket. Besides, the present study has some dissimilarities from the study by Wu et al. [21], like the implant size, the time of placement (immediate placement versus healed ridge placement), and the deviation measure method (CBCT versus scanning).

In the in vitro study by El Kholy et al. [20], it was found that in cases of a single tooth gap, using short surgical guides that covered four neighboring teeth resulted in an accuracy level equivalent to that of full-arch surgical guides covering seven teeth. This finding suggests that utilizing four teeth, with two on each side of the single tooth gap for guide support, could become the standard length for surgical guides. However, it is essential to note that this finding specifically applied to surgical guides for healing ridges. In the same study, implants placed in extraction sockets exhibited 50% higher mean platform and apex 3D deviation values and almost twice the mean angular deviation compared to implants placed in healed sites. Therefore, the results of our study indicate that increasing the number of supporting teeth beyond four could enhance the precision of immediate implant placement.

Deviation in the buccal direction can significantly impact buccal bone recession, affecting both esthetic and functional outcomes. Conversely, mesiodistal deviation can encroach upon nearby anatomical structures, such as the incisive nerve canal and adjacent roots. Therefore, assessing the risk of misalignment in both mesiodistal and buccolingual directions is crucial. A study by Chen et al. [28] indicated a preference for facial placement of implants, while other linear deviations showed no specific directional tendency, which aligns with the findings of this study. It is important to note the challenge of maintaining a central and parallel position with the drill key during implant site preparation in socket sites. Even with the surgical guide, the drills and the implant tend to move toward the least resistant space, the extraction socket, in the facial direction. In the six-teeth group, the absolute value of buccopalatal deviation was statically lower compared to the four-teeth group. This suggests that increasing the support for surgical guides could lead to more precise implant placement in immediate implants, particularly when dealing with the extraction socket located buccal to the planned implant position.

This study has several limitations: (1) the models were made of acrylic resin, which may not fully replicate human bone densities and could lead to altered results when placing implants in human patients; (2) only two surgical guide designs were compared, and there was no control group utilizing an entire arch of remaining teeth for support; (3) only one implant length was used; and (4) in vitro studies do not replicate all clinical factors that may affect implant placement accuracy, as the guide is more stable due to the absence of the tongue and oral muscles, as well as the lack of saliva, blood, and patient movement [25]. These limitations are primarily a result of the in vitro study design; therefore, further studies with a greater variety of surgical guide designs and clinical studies are needed to compare the impact of the number of supporting teeth on implant accuracy to confirm the results of the present study.

## Conclusion

In conclusion, this study offers insights into the influence of the quantity of surgical guide support on the accuracy of immediate implant placement in the maxillary anterior region. Our findings indicate that increasing the number of supporting teeth from four to six adjacent teeth significantly enhances implant placement accuracy, as measured by angular and three-dimensional deviations. Specifically, the six-teeth support group exhibited lower deviations at both the implant platform and apex, affirming the method’s improved precision. This study provides evidence that surgical guides supported by a greater number of adjacent teeth can mitigate the tendency of the implant to deviate towards areas of lesser resistance, such as extraction sockets. To enhance clinical relevance, we underscore the importance of considering guide design to optimize implant accuracy, particularly in esthetically critical areas. Future studies may explore different anatomical locations, guide designs, and varying implant lengths to validate these findings further and expand their applicability in clinical practice.

## Data Availability

The data supporting the findings of this study are available upon request from the corresponding author.
